# A behavioral test battery for mouse models of Angelman syndrome: a powerful tool for testing drugs and novel *Ube3a* mutants

**DOI:** 10.1186/s13229-018-0231-7

**Published:** 2018-09-14

**Authors:** Monica Sonzogni, Ilse Wallaard, Sara Silva Santos, Jenina Kingma, Dorine du Mee, Geeske M. van Woerden, Ype Elgersma

**Affiliations:** 1 Department of Neuroscience, Erasmus Medical Center, Rotterdam, Netherlands; 2 ENCORE Expertise Center for Neurodevelopmental Disorders, Erasmus Medical Center, Rotterdam, Netherlands

**Keywords:** Angelman syndrome, UBE3A, Mouse model, behavior, drug screening

## Abstract

**Background:**

Angelman syndrome (AS) is a neurodevelopmental disorder caused by mutations affecting UBE3A function. AS is characterized by intellectual disability, impaired motor coordination, epilepsy, and behavioral abnormalities including autism spectrum disorder features. The development of treatments for AS heavily relies on the ability to test the efficacy of drugs in mouse models that show reliable, and preferably clinically relevant, phenotypes. We previously described a number of behavioral paradigms that assess phenotypes in the domains of motor performance, repetitive behavior, anxiety, and seizure susceptibility. Here, we set out to evaluate the robustness of these phenotypes when tested in a standardized test battery. We then used this behavioral test battery to assess the efficacy of minocycline and levodopa, which were recently tested in clinical trials of AS.

**Methods:**

We combined data of eight independent experiments involving 111 *Ube3a* mice and 120 wild-type littermate control mice. Using a meta-analysis, we determined the statistical power of the subtests and the effect of putative confounding factors, such as the effect of sex and of animal weight on rotarod performance. We further assessed the robustness of these phenotypes by comparing *Ube3a* mutants in different genetic backgrounds and by comparing the behavioral phenotypes of independently derived *Ube3a*-mutant lines. In addition, we investigated if the test battery allowed re-testing the same animals, which would allow a within-subject testing design.

**Results:**

We find that the test battery is robust across different *Ube3a*-mutant lines, but confirm and extend earlier studies that several phenotypes are very sensitive to genetic background. We further found that the audiogenic seizure susceptibility phenotype is fully reversible upon pharmacological treatment and highly suitable for dose-finding studies. In agreement with the clinical trial results, we found that minocycline and levodopa treatment of *Ube3a* mice did not show any sign of improved performance in our test battery.

**Conclusions:**

Our study provides a useful tool for preclinical drug testing to identify treatments for Angelman syndrome. Since the phenotypes are observed in several independently derived *Ube3a* lines, the test battery can also be employed to investigate the effect of specific *Ube3a* mutations on these phenotypes.

## Background

Angelman syndrome (AS) is a neurodevelopmental disorder first described in 1965 by Harry Angelman, with a birth incidence of approximately 1:20,000 [1]. AS is caused by the functional loss of the maternal allele encoding an E3 ubiquitin-protein ligase (UBE3A) [2]. Loss of functional UBE3A results in the core phenotypes of severe intellectual disability, motor coordination deficits, absence of speech, and abnormal EEG, as well as in high comorbidity of sleep abnormalities, epilepsy, and phenotypes related to autism spectrum [3].

Currently, only symptomatic treatments are available for AS, primarily aimed at reducing seizures and improving sleep [4]. The development of targeted treatments for AS heavily relies on the ability to test the efficacy of treatments in mouse models of the disorder. The success of such translational studies depends on three critical factors [5]: (1) high construct validity, (2) high face validity, and (3) robustness of the behavioral phenotypes. First, the construct validity (shared underlying etiology between mouse models and patients) of the AS mouse model is very good, since AS mouse models recapitulate the patient genetics by carrying a mutated *Ube3a* gene specifically at the maternal allele. However, it should be noted that the majority of the AS patients carry a large deletion (15q11-15q13) which encompasses also other genes besides the *UBE3A* gene, and which may contribute to a more severe phenotype [6]. Second, with respect to face validity (i.e., similarity of phenotypes between patient and the mouse model), the AS mouse model captures many neurological key features of the disorder really well (e.g., epilepsy, motor deficits, abnormal EEG), as well as some of the behavioral abnormalities (abnormal sleep patterns, increased anxiety, repetitive behavior) [7–12]. Robustness of the behavioral phenotypes is the third important aspect to identify novel treatments, as it allows experiments to be sufficiently powered to detect the effect of the treatment, and meanwhile minimizes a type I error in which a drug is declared effective whereas it is not. Robustness, as well as face validity, also takes into account the sensitivity to genetic background and the extent in which a phenotype is also observed in independently derived mouse models. Notably, almost all behavioral testing described in literature has been performed using the original *Ube3a*^*tm1Alb*^ mouse strain generated in the Beaudet lab [7–9]; hence, it is unknown to what extent the reported phenotypes are actually specific to this mouse line.

We previously developed a series of behavioral paradigms in the domains of motor performance, anxiety, repetitive behavior, and seizure susceptibility, for testing the effect of *Ube3a* gene reinstatement in the inducible *Ube3a*^*mSTOP/p+*^ (*Ube3a*^*tm1Yelg*^) mice [13]. Here, we used these paradigms in a highly standardized way, to assess phenotypes in the independently derived *Ube3a*^*tm1Alb*^ and *Ube3a*^mE113X/p+^ (*Ube3a*^*tm2Yelg*^) maternal knockout strains. We combined data of eight independent experiments across five experimenters involving 111 *Ube3a*^*tm1Alb*^ and 120 wild-type littermate control mice. Using a meta-analysis, we determined the statistical power of the different behavioral tests and the effect of putative confounding factors, such as the effect of sex differences. We further assessed the robustness of these phenotypes by comparing *Ube3a* mutants in different genetic backgrounds. Finally, we employed this behavioral test battery to reassess the efficacy of minocycline and levodopa in the AS mouse model. Minocycline is a matrix metalloproteinase-9 inhibitor (MMP9), a tetracycline derivative which possesses antibiotic as well as neuroprotective activity [14, 15]. Its antibiotic properties against both gram-positive and gram-negative bacteria are related to its ability to bind to the bacterial 30S ribosomal subunit, thereby inhibiting protein synthesis [14].

Levodopa is the precursor of dopamine and was shown to be effective in treating Parkinsonism in two adults with Angelman syndrome [16]. Moreover, it is able to reduce CAMK2 phosphorylation [17], which was shown to be increased in a mouse model for Angelman syndrome [18, 19]. Minocycline and levodopa were previously tested in the AS mouse model and based on the favorable outcome of these preclinical experiments, three clinical trials were performed [20–22]. Unfortunately, none of these drugs showed a significant improvement in AS patients.

## Methods

### Mouse husbandry and breeding

For this study, we used *Ube3a*^*m−/p+*^ mice (*Ube3a*^*tm1Alb*^; MGI 2181811) [7] and *Ube3a*^*mE113X/p+*^ mutants (*Ube3a*^*tm2Yelg*^; MGI5911277) as previously described [23]. *Ube3a*^*tm1Alb*^ mice were maintained (> 40 generations) in the 129S2 background (full name: 129S2/SvPasCrl) by crossing male *Ube3a*^*m+/p−*^ mice with female 129S2 wild-type mice. *Ube3a*^*tm2Yelg*^ mice were maintained (> 20 generations) in the C57BL/6J (Charles River) background by crossing male *Ube3a*^*m+/pE113X*^ mice with female C57BL/6J wild-type mice. For the seizure susceptibility experiments with *Ube3a*^mE113X/p+^ animals, this line was backcrossed eight times in 129S2 by crossing *Ube3a*^pE113X/m+^ males with 129S2 wild-type females.

For behavioral experiments, female *Ube3a*^*tm1Alb*^ (*Ube3a*^*m+/p−*^) mice were bred to yield *Ube3a*^*m−/p+*^ mice in two different backgrounds: *Ube3a*^*m−/p+*^ (AS) mice and their WT littermates in the F1 hybrid 129S2-C57BL/6J background (WT = 120, AS = 111) and in the 129S2 background (WT = 11, AS = 16). *Ube3a*^mE113X/p+^ mice and their WT littermates were generated in the same manner in the F1 hybrid 129S2-C57BL/6J background (WT = 10, *Ube3a*^mE113X/p+^ = 10) and in C57BL/6J background (WT = 15, *Ube3a*^mE113X/p+^ = 16).

For the seizure susceptibility test, we used *Ube3a*^*m−/p+*^ (WT = 45, AS = 114) and *Ube3a*^mE113X/p+^ mice (WT = 4, AS = 8) in the 129S2 background.

Mice were housed in individually ventilated cages (IVC; 1145T cages from Techniplast) in a barrier facility. Mice were genotyped when they were 4–7 days old and re-genotyped at the completion of the experiments. All animals were kept at 22 ± 2 °C with a 12-h dark and light cycle and were tested in the light period, provided with mouse chow (801727CRM(P) from Special Dietary Service) and water ad libitum*.* During behavioral testing, mice were group-housed with two to four animals of the same sex per cage. Fighting between males was observed a few times, and in these rare cases, mice were separated and single housed. This was not a reason for exclusion. All mice were single housed during nest building and for the subsequent forced swim test. All animal experiments were conducted in accordance with the European Commission Council Directive 2010/63/EU (CCD approval AVD101002016791).

### Behavioral analysis

The weight of the animals was determined a few days before the start of the behavioral analysis. Prior to each test, mice were acclimatized to the testing room for 30 min.

All behavioral experiments were performed during the light period of the light/dark cycle. Both male and female mice at the age of 8–12 weeks were used for the experiments. Moreover, we tried to obtain a similar ratio of females/males between the WT and AS groups. Only in the experiments described in Fig. 4 (*Ube3a*^*E113X*^ mice in F1 background) and in the epilepsy experiment using *Ube3a*^*E113X*^ mice (Fig. 6c), the female/male ratio between the groups was significantly different (*p* < 0.05; chi-square test).

All behavioral testing and scoring was performed by experimenters who were blind to genotype and treatment. Behavioral tests were always run in the following order and with a minimal number of days between tests: (1) accelerating rotarod test for 5 consecutive days performed at the same hour every day; (2) 2 days of pause; (3) open field test; (4) 1 day of pause; (5) marble burying test; (6) between 5 and 7 days of pause to allow adaptation to being single caged; (7) nest building test for 5 consecutive days, in which the weight of the nest was assessed at the same hour every day; (8) 2 days of pause; and (9) forced swim test.

#### Accelerating rotarod

Motor function was tested using the accelerating rotarod (4–40 rpm, in 5 min; model 7650, Ugo Basile Biological Research Apparatus, Varese, Italy). Mice were given two trials per day with a 45–60-min inter-trial interval for 5 consecutive days (same hour every day). For each day, the average time spent on the rotarod was calculated, or the time until the mouse made three consecutive wrapping/passive rotations on the rotarod (latency in seconds). These passive rotations were observed rarely (1–2%) in 129S2 or F1 hybrid 129S2-C57BL/6J mice but rather common in (30%) C57BL/6J mice. Maximum duration of a trial was 5 min.

#### Open field test

To test locomotor activity and anxiety, mice were individually placed in a 110-cm-diameter circular open field and allowed to explore for 10 min. The light intensity was approximately 25–30 lx measured in the center of the arena. The total distance moved by each mouse in the open arena was recorded by an infrared camera (Noldus® Wageningen, NL) connected to the EthoVision® software (Noldus® Wageningen, NL), and the final outcome is indicated as distance moved in meters. For some groups, we also analyzed the time spent in the inner zone (IZ), middle zone (MZ), and outer zone (OZ) (IZ *r* = 25 cm, MZ *r* = 40, OZ *r* = 55 cm).

#### Marble burying test

Open Makrolon (polycarbonate) cages (50 × 26 × 18 cm) were filled with 4 cm of bedding material (Lignocel® Hygenic Animal Bedding, JRS). On top of the bedding material, 20 blue glass marbles were arranged in an equidistant 5 × 4 grid and the animals were given access to the marbles for 30 min. After the test, the mice were gently removed from the cage. Marbles covered for more than 50% by bedding were scored as buried, and the outcome measured is the number of buried marbles.

#### Nest building test

To measure nest building, mice were single housed for a period of 5 to 7 days before the start of the experiment. Subsequently, used nesting material was replaced and 11 g (11 ± 1) of compressed extra-thick blot filter paper (Bio-rad©) was added to the cage. The amount of the unused nest material was weighed and noted every day for a consecutive of 5 days, each day at the same hour.

#### Forced swim test

Mice were placed for 6 min in a cylindrical transparent tank (27 cm high and 18 cm diameter), filled with water (kept at 26 ± 1 °Celsius) 15 cm deep. The mouse was first left in the cylinder for 2 min to habituate. Immobility during the forced swim test was scored manually (stop-watch) by timing the amount of time the mouse was floating in the water (defined by lack of any movement) and was assessed during the last 4 min of the test. The mouse was considered to be immobile when he ceased to move altogether, making only movements necessary to keep its head above water. The outcome measured is the time in seconds in which the mouse was immobile.

#### Susceptibility to audiogenic seizures

Because of the different genetic background requirements, an independent cohort of mice was used to test susceptibility to audiogenic seizures. Mice were placed in Makrolon (polycarbonate) cages (50 × 26 × 18 cm), and audiogenic seizures were induced by vigorously scraping scissors across the metal grating of the cage lid (which creates approximately a 100-dB sound). This noise was generated for 20 s, or less if a tonic-clonic seizure developed before that time. Susceptible mice responded with wild running and leaping followed by a tonic-clonic seizure, which typically lasted 10–20 s.

#### Within-subject testing

For the experiment described in Fig. 3, *Ube3a*^*tm1Alb*^ mice in F1 hybrid 129S2-C57BL/6J background were subjected to the behavioral test battery for a second time. Once the first battery was completed, female mice that had been single housed for the nest building test were placed back together with the original cage mates, while male mice remained separated for the entire second set of behavioral tasks. The second test started 4 weeks after the first testing was completed.

### Drug administration

#### Vehicle treatment

All animals used for the meta-analysis were treated with vehicle either by IP injection (max volume 10 ul/g, hypodermic-needle 25G ×  16 mm (Sterican®/B-Braun)), by oral gavage (max 10 ul/g, stainless steel animal feeding tubes 20G ×  38 mm (Instech Laboratories)), or by adding to the drinking water.

#### Minocycline treatment

The adult-treated group consisted of 8–10-week-old *Ube3a*^*m−/p+*^ (*n* = 11 saline; 11 minocycline) and WT (*n* = 9 saline; 10 minocycline) littermate control mice in F1 hybrid 129S2-C57BL/6J background. Due to space limitations, only six animals per group were used for nest building. Mice were assigned to two treatment groups in such a way that both groups had a comparable distribution of males and females and mutant and wild-type mice. Mice were subjected to daily minocycline or vehicle IP injections (minocycline hydrochloride, Sigma-Aldrich 45 mg/kg in saline solution), starting 3 weeks prior to commencing behavioral testing, as previously described [20, 24]. Behavioral testing was started 1.5 h post-injection, based on the half-life of minocycline (~ 2 h in plasma), and the peak brain levels are reached about 2 h after injection [25].

For the postnatal-treated group, cages with *Ube3a*^*m−/p+*^ and WT pups in F1 hybrid 129S2-C57BL/6J background were split in two treatment groups in such a way that both groups had a comparable distribution of males and females and mutant and wild-type mice. The treatment group received minocycline via the lactating dam, which received minocycline through the drinking water (0.2 mg minocycline/ml, supplemented with 1 mg/ml aspartame to counteract the bitter taste and shielded for light) [26]. This method of administration was shown to yield detectable concentration of minocycline in the blood of adult mice [27] and in the breast milk of lactating dams [28, 29]. Once the mice were weaned, they were supplied with the same concentration of minocycline in their drinking water. Assuming a water intake of 1.5 ml/10 g body weight/day [30], and assuming an average weight of 25 g/mouse, the average amount of minocycline these mice received is approximately 30 mg/kg/day. The drinking water was refreshed every other day. Treatment continued until all behavioral experiments were completed. The control group received water with aspartame.

#### Levodopa/carbidopa treatment

Cages containing *Ube3a*^*m−/p+*^ and wild-type littermate control mice (8–12 weeks old) in the F1 hybrid 129S2-C57BL/6J background were assigned to two treatment groups in such a way that both groups had 15 wild type and 15 mutants and a comparable distribution of males and females. Mice in the treatment group received 15 mg/kg levodopa and 3.75 mg/kg carbidopa dissolved in saline (levodopa, Sigma-Aldrich; carbidopa, Sigma-Aldrich) by IP injection with an injection volume of 10 ul/g. The untreated group received vehicle injection by IP as described by Tan et al. [21]. The mice were injected 1 h prior to carrying out the behavioral tasks, during the entire period while partaking in these tests.

#### Levetiracetam treatment

*Ube3a*^*m−/p+*^ mice in the 129S2 background were first tested for audiogenic seizure susceptibility at baseline. Minimally 24 h later, the mice were again tested for audiogenic seizure susceptibility, this time precisely 1 h following a single IP injection of levetiracetam (0–0.5–1–2–10–15 mg/kg; Sigma-Aldrich). The injection volume used is 5 ml/kg, and the drug was dissolved in 1% Tween-80 (Sigma-Aldrich) in milliQ water as previously described [31].

### Data analysis

Data was analyzed using Excel 2010 (Microsoft) and IBM SPSS software (NY, USA). The open field, marble burying, and forced swim test data were analyzed using an unpaired *T* test in the untreated experimental groups and a two-way ANOVA in minocycline- and levodopa-treated animals (in which we assessed a genotype-treatment interaction). Rotarod and nest building were measured with a repeated measures ANOVA in the untreated experimental groups, or with a multivariate repeated measures ANOVA (assessing significance of interaction of time, genotype, and treatment) in the minocycline and levodopa experimental groups. We used a *Bonferroni’s* post hoc *test* to detect significant differences in male and female groups. For the within-subject experiment, we used a paired *T* test for open field, marble burying, and forced swim tests, while we used a repeated measures factorial ANOVA when analyzing the rotarod and the nest building test. For the audiogenic seizure analysis, a Fisher’s exact test was used. The correlation between body weight and maximal performance on the rotarod test was assessed with a Pearson’s correlation test. For the power calculation, we performed a priori analysis using G^*^Power 3.1 software [32] with *α* = 0.05 and power (1 − *β*) = 0.95, 0.90, or 0.80. Data is presented as mean± SEM in all figures. For all tests, statistical significance was denoted by *p* ≤ 0.05 (*), *p* < 0.01 (**), and *p* < 0.001 (***).

A chi-square test was performed to test if there were any significant differences in the ratio of females/males between the WT and AS groups.

## Results

### Robust behavioral phenotypes in *Ube3a*^*m−/p+*^ mice in the F1 hybrid 129S2-C57BL/6J background

**Fig. 1:**
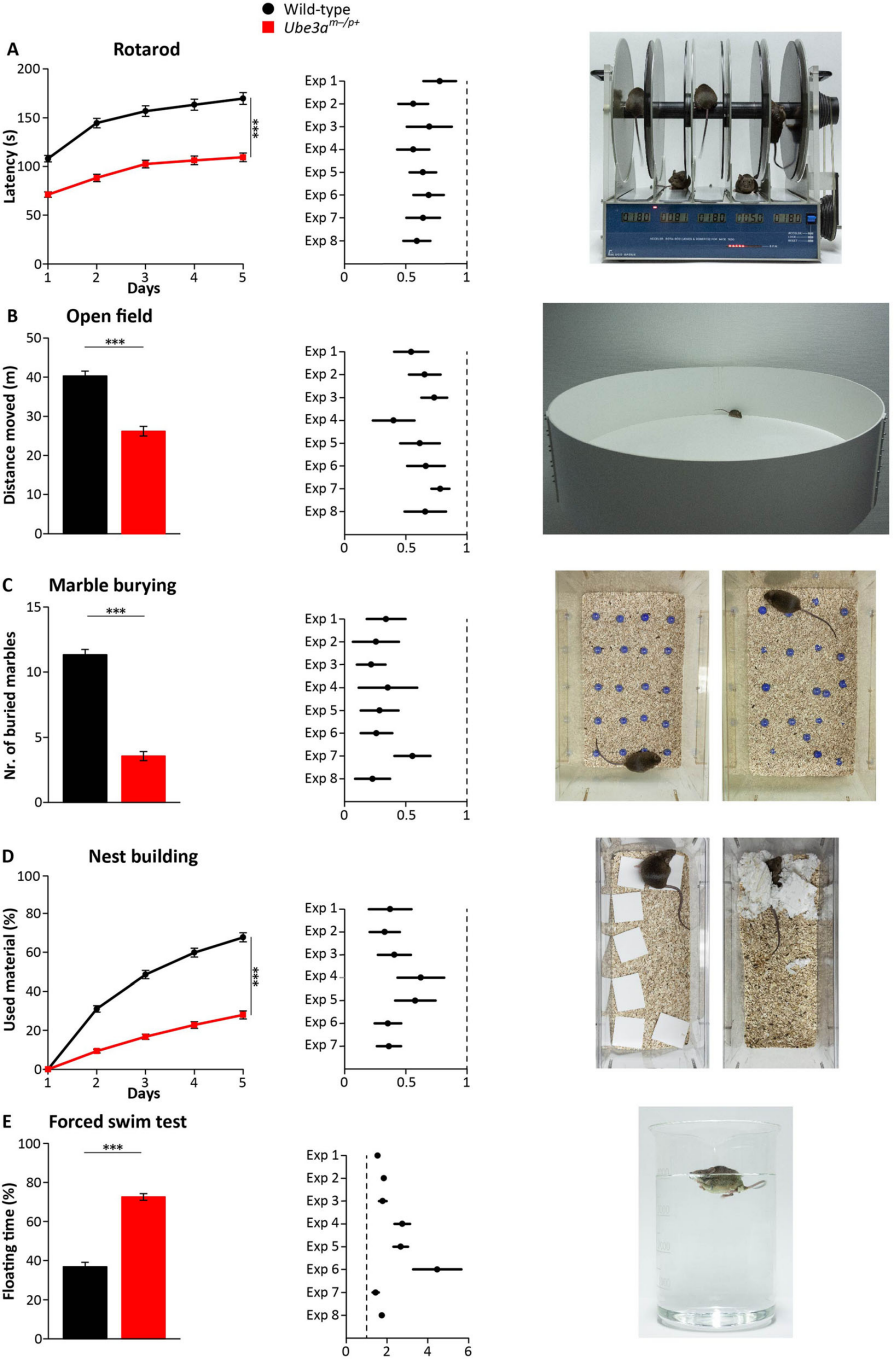
Behavioral testing of *Ube3a*^*tm1Alb*^ mice in F1 hybrid 129S2-C57BL/6J background. For each behavioral paradigm, the pooled (raw) data of all experiments is presented on the left panel, whereas the Forrest plots in the middle panel show the normalized data of the individual experiments (in which the data of each experiment is normalized against wild type; represented by a dashed line), as well as the 95% confidence interval. The picture on the right panel shows the behavioral set-up used for our experiments. For the marble burying test and nest building test, the picture shows the onset and finish of a behavioral experiment. **a** Accelerating rotarod in wild-type (WT) and *Ube3a*^*m−/p+*^ mice (*n* = 120, 111). **b** Open field test in WT and *Ube3a*^*m−/p+*^ mice (*n* = 113, 106). **c** Marble burying test in WT and *Ube3a*^*m−/p+*^ mice (*n* = 120, 111). **d** Nest building test in WT and *Ube3a*^*m−/p+*^ mice (*n* = 94, 86). **e** Forced swim test in WT and *Ube3a*^*m−/p+*^ mice (*n* = 120, 111). All data represent mean ± SEM. A repeated measures ANOVA or *T* test was used for statistical comparison of the non-normalized data. All tests show a significance effect of genotype (***p < 0.001)

We recently developed a number of behavioral tests for testing the effect of gene reinstatement in inducible *Ube3a*^*mSTOP/p+*^ (*Ube3a*^*tm1Yelg*^) mice [13]. These tests can be applied in successive order to assess phenotypes in the domains of motor performance, anxiety, and repetitive behavior. Here, we set out to assess the robustness of these phenotypes in an independently derived mouse model of AS, by using F1 hybrid 129S2-C57BL/6J *Ube3a*^*m−/p+*^ (*Ube3a*^*tm1Alb*^) mice [7], which is the *Ube3a* mouse mutant used for nearly all behavioral studies. We have frequently used this strain to test the efficacy of novel treatments and combined all data obtained from vehicle-treated *Ube3a*^*m−/p+*^ and wild-type littermate controls in the F1 hybrid 129S2-C57BL/6J background to perform a meta-analysis. In total, this constitutes the combined data of eight experiments, carried out by five experimenters and totaling 111 *Ube3a*^*m−/p+*^ and 120 wild-type littermate controls (Table 1; Fig. 1).

**Table 1:** Overview of experiments used for the meta-analysis

Exp. #	Person	WT/MUT (*n*)	Sex WT f/m MUT f/m (*n*)	Rotarod (time(s)) WT mean (SD) Mut mean (SD)	Open field (distance(m)) WT mean (SD) Mut mean (SD)	Marble burying (# marbles buried) WT mean (SD) Mut mean (SD)	Nest building (% material used) WT mean (SD) Mut mean (SD)	Forced swim test (% floating) WT mean (SD) Mut mean (SD)
1	A	15/13	8/7 6/7	128 (42) 96 (32)	41 (14) 22 (11)	11 (4) 4 (3)	14 (25) 79 (18)	53 (23) 83 (7)
2	A	15/13	5/10 3/10	142 (43) 80 (32)	49 (10) 32 (12)	8 (4) 2 (3)	36 (23) 79 (14)	44 (24) 81 (7)
3	A	15/13	5/10 3/10	133 (42) 92 (46)	40 (12) 29 (8)	11 (3) 2 (2)	27 (18) 70 (18)	41 (19) 73 (14)
4	B	21/17^1)^	10/11 7/10	159 (60) 102 (36)	31 (12) 19 (11)	14(4) 4(5)	10 (11) 48 (25)	24 (22) 63 (18)
5	C	9/11^2)^	5/4 6/5	163 (49) 91 (37)	25 (6) 10 (7)	12 (5) 4(5)	48 (27) 69 (12)	28 (24) 76 (8)
6	D	15/14^3)^	4/11 6/8	107 (44) 74 (26)	44 (7) 29 (13)	12 (3) 3 (3)	40 (18) 79 (12)	14 (20) 60(31)
7	E	15/15	8/7 7/8	196 (57) 126(52)	45(10) 35 (7)	11 (5) 6 (3)	63 (20) 74 (14)	47 (20) 67 (14)
8	A	15/15^4)^	7/8 8/7	162 (49) 95 (35)	49 (9) 33 (13)	10 (3) 2 (3)	N/A	47 (20) 88 (9)

All experiments were performed using *Ube3a*^*tm1Alb*^ mice in F1 hybrid 129S2-C57BL/6J background. For all tests shown in this table, we found a significant effect of genotype (*p* < 0.05), except for the nest building test of experiment 8, which was not performed. The table indicates the individual that performed the test battery, the number of wild-type and mutant mice used for each test, the number of females and males used for each experimental group, and the mean and standard deviation of the outcomes obtained. For the rotarod, we indicated the average performance over the 5 days, while for the nest building we provided the data as measured at day 5. Note that for some of the tests, we used a different number of mice (mice were not properly tracked, or a smaller cohort was used for nest building because of space limitations). The adapted *n* for these experiments is as follows: ^1)^nest building 13/12, forced swim test 20/17; ^2)^nest building 6/7; ^3)^open field 13/14; ^4)^open field 10/10, nest building not performed

Individuals with Angelman syndrome show clear motor impairments, and impaired performance on the accelerating rotarod is the most frequently described phenotype in *Ube3a* mice. Indeed, our meta-analysis shows a very robust significant difference between the two genotypes (*p* < 0.001; Fig. 1a). A power analysis with *α* = 0.05; (1 − *β*) = 0.95 showed that this task requires 14 animals per genotype (Table 2).

**Table 2:** Achieved power for each behavioral test of the behavioral test battery

	Wild type (mean ± SD)	Ube3a (mean ± SD)	Test	Achieved effect size	Sample size per group *(1-β)*= 0.95	Sample size per group *(1-β)* = 0.90	Sample size per group *(1-β)* = 0.80
Rotarod Time on machine (s)	149 ± 55	95 ± 40	ANOVA	0.56	14	11	9
Open field Distance moved (m)	40 ± 13	26 ± 13	*T* test	1.17	21	17	13
Marble burying (# marbles buried)	11 ± 4	4 ± 4	*T* test	2.26	7	6	5
Nest building (% used nesting material)	68 ± 23	28 ± 19	*T* test	1.95	8	7	6
Forced swim test (% floating time)	37 ± 25	73 ± 18	*T* test	1.73	10	9	7
Susceptibility to audiogenic seizure (% of animals)	7	98	*T* test	4.55	3	3	3

Data provided is based on the experiments using *Ube3a*^*tm1Alb*^ mice in F1 hybrid 129S2-C57BL/6J background. The table provides the obtained effect size, number of mice needed per genotype for each behavioral test (with power equal to 0.95, 0.90, 0.80), and statistical test used. For rotarod calculations, we used the average performance over the 5 days, while for the nest building we used the data of the last test day

Following 2 days of rest, the same mice were then tested in the open field test. This paradigm is commonly used to assess anxiety in mice. Increased anxiety is commonly observed in individuals with AS [33], as well as individuals with autism spectrum disorder. In this test, we place the mice in an open arena situated in a brightly lit room and record the distance the mice travel during a 10-min time span. The measurements of the distance moved in the open arena indicated that AS mice moved significantly less (WT 40.3 ± 1.2 m; AS 26.2 ± 1.2 m; *p* < 0.001; Fig. 1b). A power analysis (α = 0.05; (1 − *β*) = 0.95) showed that this task requires a minimum number of 21 mice per genotype, which makes this test a relative weak test (Table 2). Previous studies reported no significant difference observed between genotypes in the time spent in the [8, 9] inner zone of the open field, which is another measure of anxiety. Our meta-analysis revealed a significant difference between genotypes (*p* < 0.005), but this difference was small (WT 1.1% versus mutant 0.7% time in inner zone), and a significant effect was only observed in four out of the eight individual experiments (data not shown).

After 1 day of rest, the same mice were then analyzed in the marble burying test, a test used to assess repetitive and perseverative behavior as well as anxiety [34, 35]. When exposed to marbles, AS mice show a strongly impaired marble burying behavior compared to WT mice (WT 11.3 ± 0.4; AS 3.6 ± 0.3; *p* < 0.001; Fig. 1c). A power analysis (*α* = 0.05; (1 − *β*) = 0.95) showed that seven animals/group are sufficient for this test, indicating a very robust phenotype (Table 2).

After the marble burying task, all mice were single housed for 5–7 days and then analyzed for 5 consecutive days while performing the nest building test. The nest building test assesses the innate behavior of mice to create a nest to maintain body temperature and to find shelter [36]. AS mice showed a clear phenotype compared to their WT control littermates (*p* < 0.001; Fig. 1d). As indicated in Table 2, the nest building phenotype is quite robust, since it only requires 8 mice (*α* = 0.05; (1 − *β*) = 0.95) per group if analyzed over the last day.

Following 2 days of pause, the animals were finally subjected to the forced swim test, in which the mouse is placed in a beaker filled with water, from which the mouse will try to escape by swimming. This test is typically used to test depressive-like behavior in mice [37]. AS mice showed significant more time floating (instead of swimming) compared to WT mice (WT 36.8 ± 2.3; AS 72.6 ± 1.7; *p* < 0.001; Fig. 1e). The power analysis test showed that this task requires a minimum of 10 mice (*α* = 0.05; (1 − *β*) = 0.95).

Taken together, the data indicates that this test battery yields a series of robust behavioral phenotypes that can be obtained in a relative quick manner using a single cohort of mice.

### The dependence of sex on the behavioral phenotypes

**Fig. 2:**
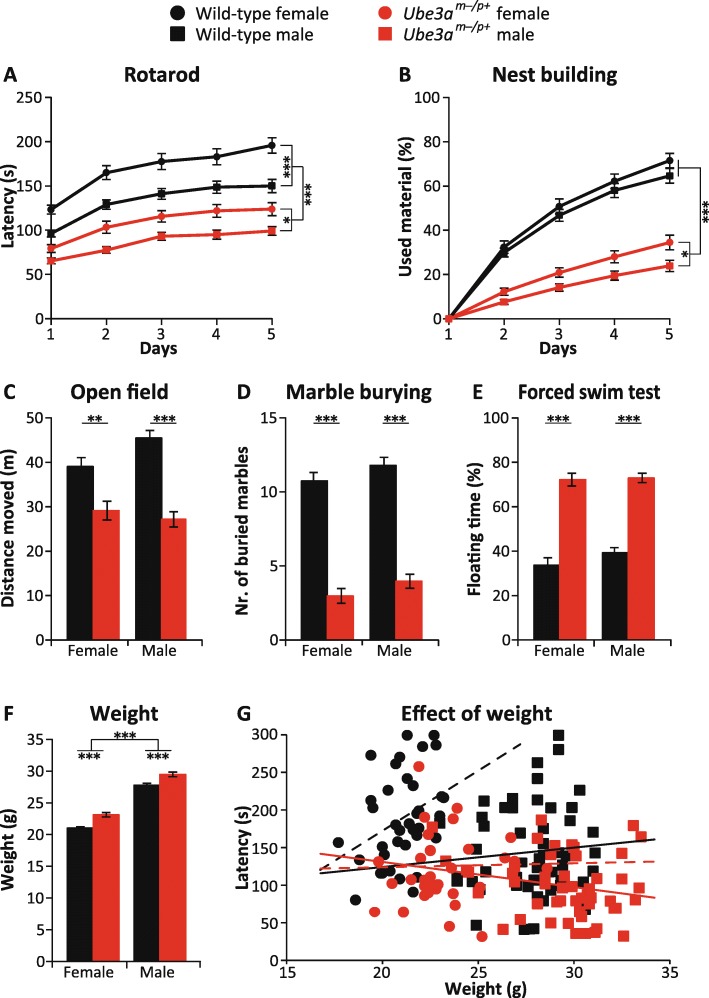
Effect of sex on the behavioral phenotypes of *Ube3a*^*tm1Alb*^ mice in F1 hybrid 129S2-C57BL/6J background. **a** Accelerating rotarod in WT and *Ube3a*^*m−/p+*^ female mice (*n* = 52, 46) and in WT and *Ube3a*^*m−/p+*^ male mice (*n* = 68, 65). **b** Nest building test in WT and *Ube3a*^*m−/p+*^ female mice (*n* = 42, 33) and in WT and *Ube3a*^*m−/p+*^ male mice (*n* = 52, 53). **c** Open field test in WT and *Ube3a*^*m−/p+*^ female mice (*n* = 47, 41) and in WT and *Ube3a*^*m−/p+*^ male mice (*n* = 66, 65). **d** Marble burying test in WT and *Ube3a*^*m−/p+*^ female mice (*n* = 52, 46) and in WT and *Ube3a*^*m−/p+*^ male mice (*n* = 68, 65). **e** Forced swim test in WT and *Ube3a*^*m−/p+*^ female mice (*n* = 52, 46) and in WT and *Ube3a*^*m−/p+*^ male mice (*n* = 68, 65). **f** Bodyweight in WT and *Ube3a*^*m−/p+*^ female mice (*n* = 37, 33) and in WT and *Ube3a*^*m−/p+*^ male mice (*n* = 53, 50). **g** Pearson correlation test between body weight and latency to fall at day 5 in WT and *Ube3a*^*m−/p+*^ female mice (*n* = 37, 33) and in WT and *Ube3a*^*m−/p+*^ male mice (*n* = 53, 50). Multivariate repeated ANOVA or a two-way ANOVA was used for statistical comparison. A *Bonferroni’s* post hoc *test* was used to detect significant differences in behavioral phenotypes of male and female groups. All data represent mean ± SEM. Significant effects of genotype or sex are indicated as *p < 0.05, **p < 0.01, and ***p < 0.001

Angelman syndrome affects both males and females, with no known differences between the sexes. To assess if this is also the case for the *Ube3a* mouse phenotypes described above, we analyzed if there were any significant sex differences. An effect of sex was noted on the rotarod, in which female wild-type and *Ube3a* mice performed significantly better than male wild-type and *Ube3a* mice (*p* < 0.001; Fig. 2a). Since male mice are heavier than female mice and since *Ube3a*^*m−/p+*^ mutants show increased weight (Fig. 2f) [8, 38], we investigated if the impaired rotarod performance as seen in *Ube3a*^*m−/p+*^ mutants could be attributed to their increased weight. Hence, we performed a correlation analysis between body weight and time on the rotarod (as measured on the last training day). As shown in Fig. 2g, no meaningful correlation is observed between body weight and latency to fall in both WT mice and AS mice (WT males Pearson *r* = 0.08, AS males Pearson *r* = − 0.21, WT females Pearson *r* = 0.35, AS females Pearson *r* = 0.02), although the correlation observed in WT female mice was just statistically significant (*p* < 0.05), indicating that increased bodyweight actually improves (rather than impairs) rotarod performance. Overall, we conclude that the impaired motor performance of *Ube3a*^*m−/p+*^ mutants on the rotarod is not caused by the increased body weight observed in these mice, but truly reflects differences in motor performance.

We also observed a small effect of sex for the nest building task in which female *Ube3a*^*m−/p+*^ mutants outperformed the male *Ube3a*^*m−/p+*^ mutants (*p* < 0.05). A similar tendency was also observed in wild-type mice, but this effect was not significant (Fig. 2b). Despite the slightly better performance of female *Ube3a*^*m−/p+*^ mutants, female *Ube3a*^*m−/p+*^ mutants were still significantly different from wild-type mice (*p* < 0.001).

We observed no significant effect of sex in the open field test (*p* = 0.25), marble burying test (*p* = 0.06), and forced swim test (*p* = 0.27; Fig. 2c–e). Overall, these data suggest that the set of behavioral phenotypes observed in AS mice are robust and are not markedly influenced by the sex of the animal. However, given the decreased performance of male mice on the rotarod, mixed cohorts used for rotarod testing should be well balanced with respect to sex to obtain a reliable phenotype.

### The behavioral test battery is suitable for within-subject testing design

**Fig. 3:**
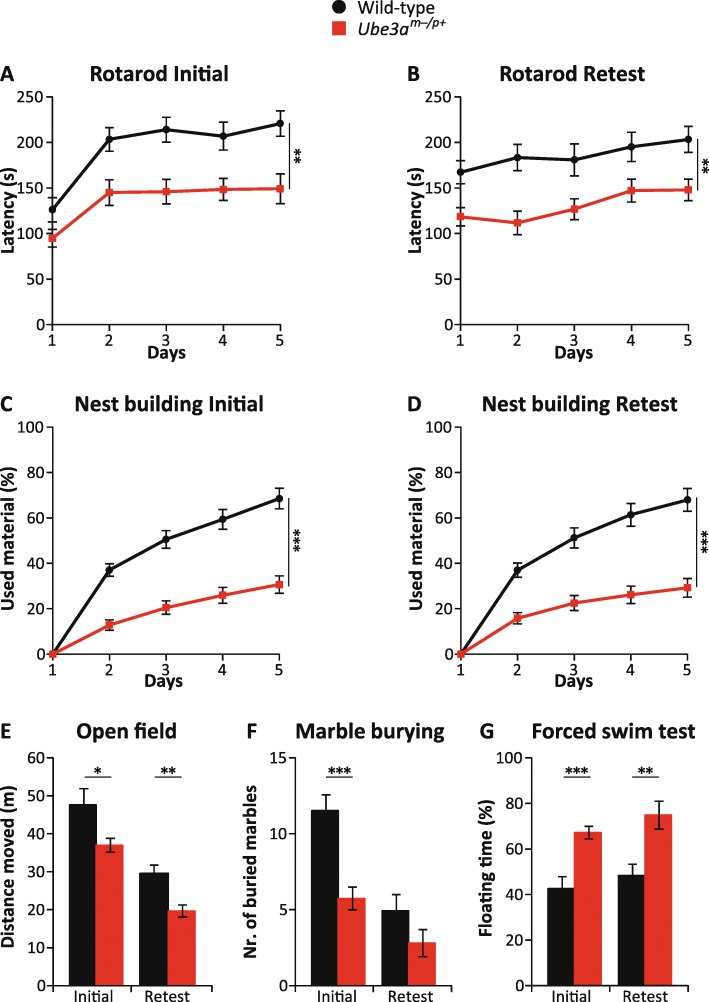
Most behavioral phenotypes are stable upon re-testing *Ube3a*^*tm1Alb*^ mice in F1 hybrid 129S2-C57BL/6J background. **a**, **c**, **e**–**g** WT and *Ube3a*^*m−/p+*^ mice at initial testing and **b**, **d**, **e**–**g** upon re-testing. A single cohort of 15 wild-type (8 females, 7 males) and 15 *Ube3a*^*tm1Alb*^ (8 females, 7 males) mice was used for all experiments. A repeated measures ANOVA or *T* test was used for statistical comparison of genotypes, as described in the legend of Fig. 1. All data represent mean ± SEM. Significant effects of genotype are indicated as *p < 0.05, **p < 0.01, and ***p < 0.001 for genotype significance

A within-subject testing design is a powerful design for drug testing purposes, as it allows assessing the efficacy of a drug with considerable fewer animals. Therefore, we investigated whether the behavioral test battery allowed re-testing the same animals while maintaining a similar phenotype, which is a prerequisite for applying a within-subject design. We subjected 15 *Ube3a*^*m−/p+*^ mice (*Ube3a*^*tm1Alb*^) and 15 WT littermates in the F1 hybrid 129S2-C57BL/6 background to the behavioral test battery and repeated the test battery after a pause of 4 weeks. As shown in Fig. 3, performance on the rotarod test, nest building test, and forced swim test was highly similar when the initial test data were compared to the re-testing data. However, performance in the open field test as well as marble burying test was significantly different when this test was performed for the second time (open field: wild type initial vs retest *p* < 0.001, *Ube3a*^*m−/p+*^ initial vs retest *p* < 0.001; marble burying: wild type initial vs retest *p* < 0.001, *Ube3a*^*m−/p+*^ initial vs retest *p* < 0.001; paired *T* test). These differences upon re-testing are likely due to the decreased anxiety levels and or habituation of the mice upon re-testing in these paradigms. Importantly, *Ube3a*^*m−/p+*^ mice remained significantly different from wild-type littermates when tested for a second time, with the exception of the marble burying test, which no longer yielded a phenotype upon re-testing (*p* = 0.13). Hence, we conclude that most tests of the behavioral test battery are suitable for a within-subject design to test the efficacy of a drug.

### Behavioral phenotypes are also observed in the *Ube3a*^*E113X*^ mouse model

**Fig. 4:**
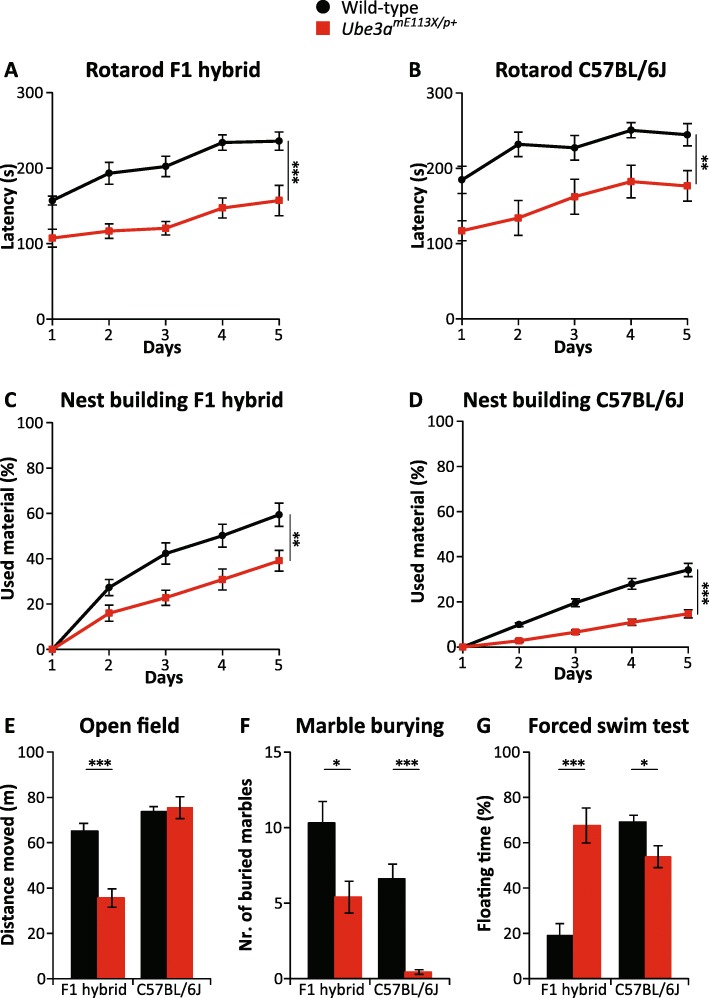
Behavioral testing of *Ube3a*^mE113X/p+^ (*Ube3a*^*tm2Yelg*^) mice in the F1 hybrid 129S2-C57BL/6J and the C57BL/6J background. **a**, **b** Accelerating rotarod in WT and *Ube3a*^mE113X/p+^ mice in F1 hybrid 129S2-C57BL/6J and C57BL/6J background. **c**, **d** Nest building test in WT and *Ube3a*^mE113X/p+^ mice in F1 hybrid 129S2-C57BL/6J and C57BL/6J background. **e**–**g** Open field, marble burying, and forced swim tests in WT and *Ube3a*^mE113X/p+^ mice in F1 hybrid 129S2-C57BL/6J and C57BL/6J background*.* For all behavioral tests, we used a single cohort of 10 wild-type (1 female, 9 males) and 10 *Ube3a*^mE113X/p+^ mice (6 females, 4 males) in F1 hybrid 129S2-C57BL/6J, and 15 wild-type (11 females, 4 males) and 16 *Ube3a*^mE113X/p+^ (*Ube3a*^*tm2Yelg*^) (13 females, 4 males) mice in C57BL/6J background. All data represent mean ± SEM. A repeated measures ANOVA or *T* test was used for statistical comparison of genotypes, as described in the legend of Fig. 1. Significant effects of genotype are indicated as *p < 0.05, **p < 0.01, and ***p < 0.001

The results above indicate that the behavioral test battery gives robust phenotypes in the *Ube3a*^*tm1Alb*^ line as well as in the previously published *Ube3a*^*mSTOP/p+*^ (*Ube3a*^*tm1Yelg*^) line. In order to test the robustness of the battery in a third independently derived *Ube3a*-mutant strain, we used the *Ube3a*^mE113X/p+^ (*Ube3a*^*tm2Yelg*^) strain, which we recently described [23]. As shown in Fig. 4, the *Ube3a*^mE113X/p+^-mutant mice in the F1 129S2-C57BL/6J background showed again clear impairments on the rotarod test (*p* < 0.001), open field test (*p* < 0.001), marble burying test (*p* < 0.05), nest building test (*p* < 0.01), and forced swim test (*p* < 0.001). Taken together, these data suggest that the identified set of behavioral phenotypes in this test battery is present in three independently derived *Ube3a*-mutant lines.

### Mouse genetic background affects the identified AS phenotypes

**Fig. 5:**
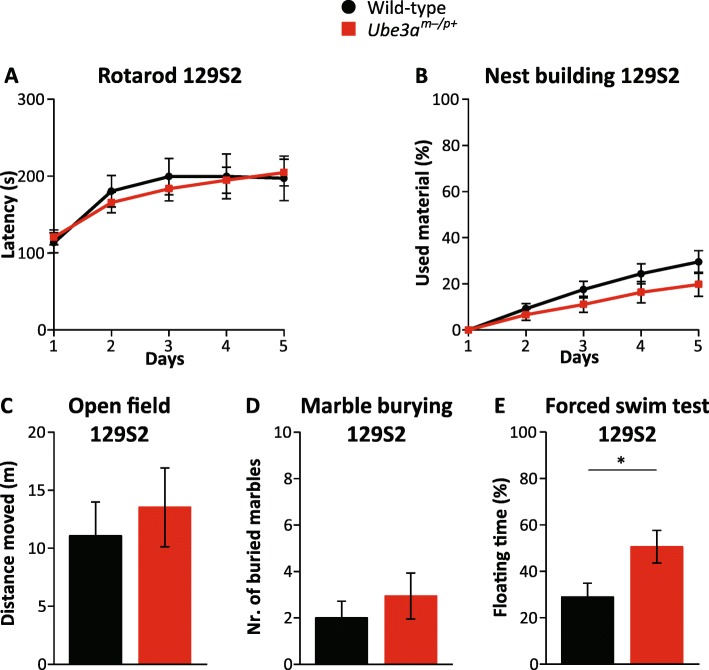
Behavioral testing of *Ube3a*^*m−/p+*^ (*Ube3a*^*tm1Alb*^) mice in the 129S2/SvPasCrl background. **a**–**e** Accelerating rotarod, nest building, open field, marble burying, and forced swim test in wild-type and *Ube3a*^*tm1Alb*^ mice in 129S2/SvPasCrl background (*n* = 11, 16) (WT = 5 females, 6 males) (*Ube3a*^*m−/p+*^ = 8 females, 8 males). A repeated measures ANOVA or *T* test was used for statistical comparison of genotypes, as described in the legend of Fig. 1. Significant effects of genotype are indicated as *p < 0.05

Previous studies have indicated the importance of the genetic background for certain *Ube3a* phenotypes [8, 9]. To test the importance of the genetic background on the behavioral test battery, we performed the test battery on AS mice on a pure C57BL/6J (Fig. 4) and 129S2 background (Fig. 5) instead of the F1 hybrid background. *Ube3a*^mE113X/p+^ mice in C57BL/6J background showed a similar phenotype as *Ube3a*^mE113X/p+^ mutants in the F1 hybrid 129S2-C57BL/6J background with respect to the rotarod test (*p* < 0.01), marble burying test (*p* < 0.001), and nest building test (*p* < 0.001) (Fig. 4). No deficit was observed in the open field test (*p* = 0.75). Notably, the *Ube3a*^mE113X/p+^ mice in C57BL/6J background showed a significant phenotype in the forced swim test (*p* < 0.05), however in the opposite direction compared to AS mice in F1 hybrid 129S2-C57BL/6J background.

The test battery was also performed using *Ube3a*^*tm1Alb*^ mice in the inbred 129S2 background (Fig. 5). *Ube3a*^*tm1Alb*^ mice in the 129S2 background did not show any of the phenotypes observed in *Ube3a*^*tm1Alb*^ mice in the F1 hybrid background, with the exception of the forced swim test (*p* < 0.05), which yielded a similar result as obtained in mice in the F1 hybrid background. Taken together, these data confirm and extend previous studies that most AS mouse phenotypes are strongly dependent on the genetic background.

### Susceptibility to audiogenic seizures

Epilepsy is a common feature of individuals with AS [39]. We previously showed that *Ube3a*^*tm1Alb*^ mice as well as *Ube3a*^*mSTOP/p+*^ (*Ube3a*^*tm1Yelg*^) mice are highly susceptible to audiogenic seizures, a phenotype that is specifically observed in mice in the 129S2 background [7]. To investigate the strength of this test in more detail, we performed a meta-analysis of five independent experiments with a total of 114 *Ube3a*^*m−/p+*^ (*Ube3a*^*tm1Alb*^) mice and 45 wild-type littermates in the 129S2 background. This analysis showed that this is a very robust phenotype with seizures observed in 98% of *Ube3a*^*m−/p+*^ mice and in 7% of the wild-type littermates (*p* < 0.001)*.* The robustness of this test was further confirmed by a power calculation analysis (Table 2).

**Fig. 6:**
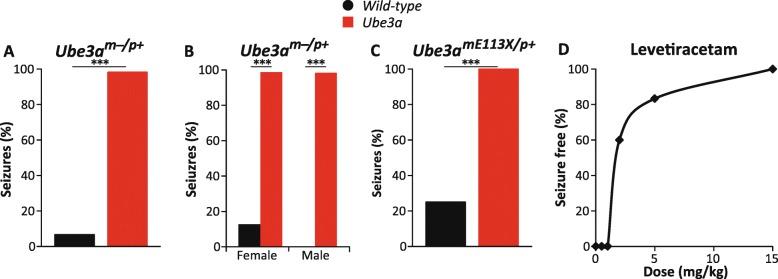
Audiogenic seizure susceptibility of *Ube3a*^*m−/p+*^ and *Ube3a*^mE113X/p+^ mice in the 129S2/SvPasCrl background. **a** Audiogenic seizure susceptibility of WT and *Ube3a*^*m−/p+*^ mice (*n* = 45, 114). **b** Effect of sex on seizure susceptibility in wild-type and *Ube3a*^*m−/p+*^ mice (females *n* = 24, 62; males *n* = 21, 52). **c** Seizure susceptibility in wild-type and *Ube3a*^mE113X/p+^ mice (*n* = 4, 8) (WT = 3 females, 1 males; *Ube3a*^mE113X/p+^ = 1 females, 7 males). **d** Effect of increasing doses of levetiracetam on epilepsy susceptibility of *Ube3a*^*m−/p+*^ mice (0 mg/kg, *n* = 12; 0.5 mg/kg, *n* = 6; 1 mg/kg, *n* = 6; 2 mg/kg, *n* = 30; 5 mg/kg, *n* = 30; 15 mg/kg, *n* = 30). Fisher’s exact test was used for statistical comparison. ***p < 0.001 for genotype significance

We tested whether seizures were also present in the *Ube3a*^mE113X/p+^ (*Ube3a*^*tm2Yelg*^) line. To that end, we crossed *Ube3a*^pE113X/m+^ females (backcrossed eight times in 129S2) with 129S2 males. As shown in Fig. 6, an audiogenic seizure could be provoked in all *Ube3a*^mE113X/p+^ mutants tested (*p* < 0.001), indicating that this phenotype is observed across three independently derived *Ube3a-*mutant lines.

We previously demonstrated that the sensitivity to audiogenic seizures can be reversed upon acute treatment with anti-epileptic drugs [13]. Given the high power of this assay, we investigated if this assay is suitable to determine the effective dose of a treatment. To that end, we treated mice with levetiracetam, a compound that acts as ligand of the synaptic vesicle protein 2A, which is a commonly used anti-epileptic drug for both partial and generalized seizures and which is also often prescribed to individuals with AS [40, 41]. *Ube3a*^*m−/p+*^ (*Ube3a*^*tm1Alb*^) mice in 129S2 background were first assessed for their (baseline) sensitivity to audiogenically evoked seizures without treatment. After establishing that all mice were sensitive, mice received at least 1 day after baseline testing a single IP dose of levetiracetam and were tested 1 h after IP injection. As shown in Fig. 6d, a good dose-response curve could be obtained, in which 2 mg/kg levetiracetam yielded approximately 60% of mice to be resistant to audiogenic seizures. This indicates that this test is highly suitable for quickly determining the effective dose of a treatment.

### Minocycline treatment does not improve behavioral phenotypes of *Ube3a* mice

**Fig. 7:**
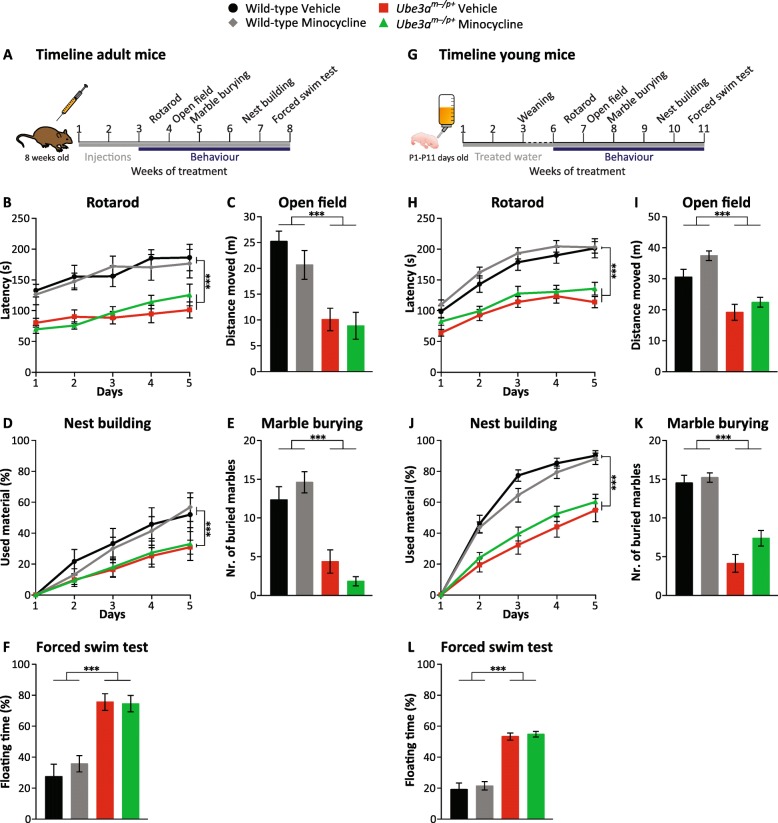
Effect of minocycline treatment on adult and young *Ube3a*^*tm1Alb*^ mice in F1 hybrid 129S2-C57BL/6J background. **a** Timeline representing minocycline treatment and behavioral phenotyping of adult *Ube3a*^*m−/p+*^ mice. **b**–**f** Effect of minocycline on adult *Ube3a*^*tm1Alb*^ mice on the behavioral test battery. Wild-type and *Ube3a*^*m−/p+*^ (*Ube3a*^*tm1Alb*^) vehicle-treated adult mice: *n* = 9, 11 (WT = 5 females, 4 males; *Ube3a*^*m−/p+*^ = 6 females, 5 males), with the exception of the nest building (*n* = 6, 7). Minocycline-treated wild-type and *Ube3a*^*m−/p+*^ (*Ube3a*^*tm1Alb*^) adult mice: *n* = 10, 11 mice (WT = 6 females, 4 males; *Ube3a*^*m−/p+*^ = 6 females, 5 males), with the exception of the nest building (*n* = 6, 6). **g** Timeline representing minocycline treatment and behavioral phenotyping of young *Ube3a*^*m−/p+*^ mice. **h**–**l** Effect of minocycline on young *Ube3a*^*tm1Alb*^ mice on the behavioral test battery. Wild-type and *Ube3a*^*m−/p+*^ (*Ube3a*^*tm1Alb*^) vehicle-treated young mice: *n* = 21, 17 (WT = 11 females, 10 males; *Ube3a*^*m−/p+*^ = 7 females, 10 males), with the exception of the nest building (*n* = 13, 12) and the forced swim test (20, 17). Minocycline-treated wild-type and *Ube3a*^*m−/p+*^ (*Ube3a*^*tm1Alb*^) young mice: *n* = 33, 22 mice (WT = 20 females, 13 males; *Ube3a*^*m−/p+*^ = 8 females, 14 males), with the exception of the open field (33, 21), the marble burying (33, 21), and the nest building (*n* = 16, 17). A multivariate repeated ANOVA or a two-way ANOVA was used for statistical comparison in behavioral phenotypes. *p < 0.05 and ***p < 0.001 indicate the effect of genotype. In none of the tests, we observed an interaction of genotype and treatment

It has previously been reported that minocycline treatment of *Ube3a* animals improves synaptic plasticity as well as motor coordination, which was the basis for an open-label study with minocycline in individuals with AS (trial register NCT01531582 and [20]), as well as a randomized controlled trial ((NCT02056665), [22]). Unfortunately, the randomized trial showed no difference between placebo and minocycline-treated individuals [22]. To test if minocycline ameliorated the *Ube3a*-mutant phenotypes in our behavioral test battery, we subjected the animals to the same treatment protocol as used for the initial mouse study [20]. Adult-treated *Ube3a*^*m−/p+*^ (*Ube3a*^*tm1Alb*^) mice and littermate controls (8–12 weeks of age) in the F1 hybrid 129S2-C57BL/6J background received daily minocycline (45 mg/kg) or control saline IP injections starting 3 weeks prior to behavioral testing. After 3 weeks of daily injections, the mice were sequentially subjected to the behavioral test battery as described above. In contrast to the previous finding (trial register NCT01531582), we did not observe a rescue on the rotarod. We also observed no effect of minocycline on any of the other tests of the behavioral battery (Fig. 7; two-way ANOVA, genotype/treatment interaction *p* > 0.08 in all tests). Notably, prolonged exposure to daily minocycline injections resulted in yellow deposits over the organs and dullness of the liver (data not shown), confirming previous studies that IP administration of minocycline is not the best choice of administration [42].

Minocycline has also been used to reverse the behavioral deficits of a mouse model of Fragile X [26, 43]. Notably, in these studies, minocycline treatment was initiated immediately after birth and provided though the drinking water. Since we previously showed that a behavioral rescue of *Ube3a* mice may also depend on the timing of treatment initiation [13], we decided to treat *Ube3a* animals immediately after birth, using the same protocol as described for FMRP mice [26]. However, also this prolonged postnatal treatment regimen did not yield a significant behavioral improvement, as none of these tests showed a significant interaction of genotype and treatment (two-way ANOVA, genotype/treatment interaction *p* > 0.16 in all tests) (Fig. 7).

### Levodopa/carbidopa treatment does not improve behavioral phenotypes of *Ube3a* mice

**Fig. 8:**
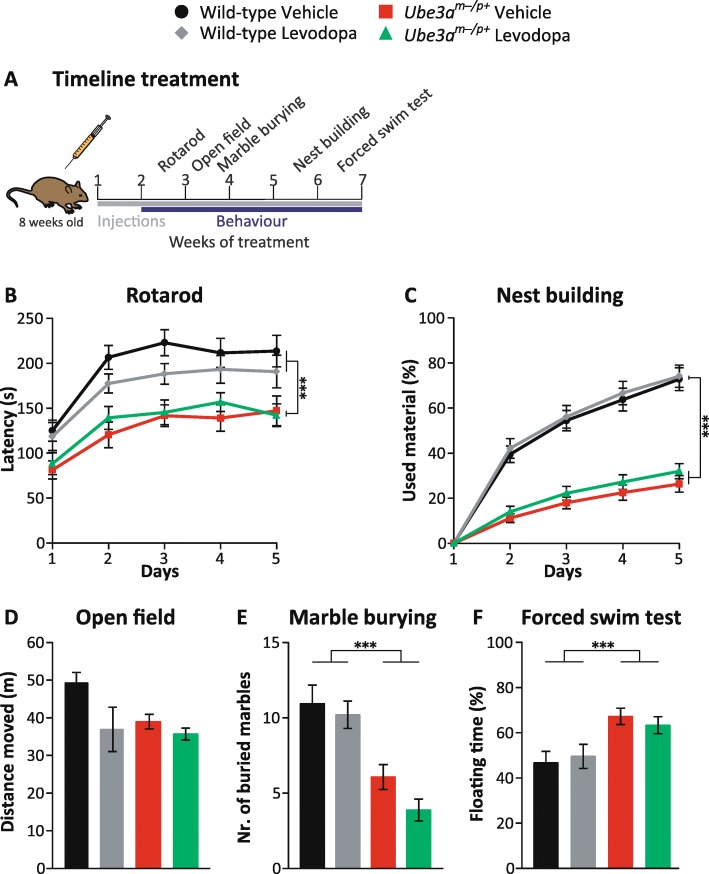
Effect of levodopa treatment on *Ube3a*^*tm1Alb*^ mice in F1 hybrid 129S2-C57BL/6J background. **a** Timeline representing levodopa treatment and behavioral phenotyping of *Ube3a*^*m−/p+*^ mice. **b**–**f** Effect of levodopa on the behavioral test battery. Wild-type and *Ube3a*^*m−/p+*^ (*Ube3a*^*tm1Alb*^) vehicle-treated mice: *n* = 15, 15 (WT vehicle = 8 females, 7 males; *Ube3a*^*m−/p+*^ vehicle = 7 females, 8 males), levodopa-treated wild-type and *Ube3a*^*m−/p+*^ (*Ube3a*^*tm1Alb*^) mice: *n* = 15, 15 mice (WT levodopa = 8 females, 7 males; *Ube3a*^*m−/p+*^ levodopa = 6 females, 9 males). A multivariate repeated ANOVA or a two-way ANOVA was used for statistical comparison in behavioral phenotypes. ***significant effect of genotype *p* < 0.001. No effect of genotype was observed in the open field test, since levodopa-treated wild-type mice were similar to *Ube3a* mice. In none of the tests, we observed an interaction of genotype and treatment

A recent study showed that treatment of *Ube3a* mice with levodopa resulted in improvement of their motor skills compared to untreated *Ube3a* mice [21]. Based on this preclinical observation, a placebo-controlled trial of levodopa was initiated in 55 children between 4 and 12 years diagnosed with AS. Unfortunately, no significant improvement was observed on any of the outcomes measured following a 1-year treatment (trial register NCT01281475 and [21]). To test as to what extent levodopa ameliorated the phenotypes of *Ube3a*^*m−/p+*^ (*Ube3a*^*tm1Alb*^) mice in our behavioral battery, we subjected the animals to the same treatment protocol as used for the initial mouse study [21]. *Ube3a*^*m−/p+*^ and wild-type littermates (8–12 weeks of age) in F1 hybrid 129S2-C57BL/6J background received daily levodopa/carbidopa (15 mg/kg levodopa and 3.75 mg/kg carbidopa) or control saline IP injections, starting 1 h prior to behavioral testing. In contrast to the earlier finding [21], we did not observe a rescue on the rotarod. We also observed no effect of levodopa treatment on any of the other tests of the behavioral battery (two-way ANOVA, genotype/treatment interaction *p* > 0.17 in all tests) (Fig. 8).

## Discussion

Robust behavioral phenotypes with high construct and face validity in mouse models of disease are critical for the identification of novel treatments and the successful translation of these therapies to clinical trials. These preclinical studies may give us important information about the therapeutic dose, optimal age of treatment, and the best outcome measures to be used in a clinical trial. Given the high failure rate of clinical trials aimed at improving cognitive function [44], it is absolutely critical that the preclinical data is robust (reproducible results across different mutant lines and different experimenters) and that the animal studies have high construct and face validity.

In this study, we investigated the robustness of a number of behavioral phenotypes, which we previously described using the inducible *Ube3a*^*mSTOP/p+*^ (*Ube3a*^*tm1Yelg*^) mice [13]. These phenotypes were assessed in two independently derived *Ube3a* lines: in the commonly used *Ube3a*^*tm1Alb*^ line [7] and the recently generated *Ube3a*^mE113X/p+^ (*Ube3a*^*tm2Yelg*^) line [13]. Recently, we have tested two additional novel *Ube3a* lines in this test battery with the same results; the *Ube3a*^*tm1.1Bdph*^ line (MGI:5882092) and a novel (unpublished) *Ube3a* line (*Ube3a*^*em1Yelg*^). Thus, taken together, a total of five independently derived *Ube3a* lines show phenotypes on all the behavioral tests of the test battery described in this study. In all cases, we used heterozygous *Ube3a* mice in which the mutation was located on the maternally inherited *Ube3a* allele. Therefore, we conclude that construct validity is very high. However, since the majority of individuals with AS carries a large chromosomal deletion of the AS critical region (15q11-q13) which encompasses also other genes besides *Ube3a* and which may contribute to a more severe phenotype [6], it would be of interest to test a mouse model of AS with large maternal deletion [11] in our behavioral test battery.

In terms of face validity, we used behavioral paradigms that assess domains of motor performance, anxiety, repetitive behavior, and seizure susceptibility, which are all relevant clinical phenotypes of AS. Nevertheless, the clinical translational value of some of our tests (e.g., open field, marble burying, nest building, and forced swim tests) may be limited. Although it is notable that many of our tests involve a strong motor component, we think that it is unlikely that the phenotypes observed in the open field, marble burying, nest building, and forced swim tests are solely related to deficits in the domain of motor functioning. Most notably, we have shown that the critical period for rescuing these phenotypes is distinctly different compared to rescuing the rotarod deficit [13] (and unpublished data). For instance, we found that gene reactivation in 3-week-old mice fully rescues the rotarod phenotype, but none of the other phenotypes [13]. It is further noticeable that both WT and mutant mice behave significantly different when tested for a second time in the open field and marble burying tests, whereas no significant changes were observed in rotarod performance. This further indicates that the deficits in the open field and marble burying tests are indicative of deficits in other domains than motor performance.

An important clinical feature of AS that is lacking in our behavioral test battery is a paradigm that assesses cognitive function. Despite profound cognitive impairments in individuals with AS, learning deficits in the AS mouse model are rather mild. We and others have reported learning deficits in AS mice by using the Morris water maze [8, 18, 45]. However, this paradigm is very labor intensive and hence less suitable for drug testing. Moreover, we found that a large number of mice are needed to detect significant differences and results varied strongly among experimenters (data not shown). A good learning paradigm that is highly suitable for drug testing is fear conditioning, in which animals are subjected to a single training session in which they are trained to associate a context (training chamber) or cue (tone) with a foot shock. However, we have not been able to get consistent results across experiments and experimenters (data not shown), and varying results are published in literature, with some studies showing a specific deficit in context conditioning [7, 46] and others a specific deficit in cued conditioning [8] or both [47–49]. Notably, the two studies that investigated the behavioral deficits of *Ube3a* mice across strains in great detail showed no context conditioning deficit in *Ube3a* mice in the F1 hybrid 129-C57BL/6J background and C57BL/6J background, and either normal [9] or impaired [8] cued fear conditioning in *Ube3a* mice in the C57BL/6J background. Collectively, these studies indicate that this phenotype is rather weak, and hence results, obtained with these tests should be interpreted with care.

By combining the data of eight independent experiments performed by five different experimenters, we were able to perform a meta-analysis of 111 *Ube3a*^*m−/p+*^ (*Ube3a*^*tm1Alb*^) and 120 WT littermate mice in the F1 hybrid 129S2-C57BL/6J background and determine the robustness of the phenotypes. In all eight experiments, we replicated *Ube3a* phenotypes observed on the rotarod test, open field test, marble burying test, nest building test, and the forced swim test. Deficits of *Ube3a* mice in rotarod performance, open field behavior, and marble burying have been reported by many other investigators, and hence, our results confirm the robustness of these tests. Impaired nest building behavior and impaired performance in the forced swim test of *Ube3a* mice have not yet been reported by other laboratories, but our study shows that these deficits are also very robust. In fact, a power analysis showed that these tests are among the most robust tests of the behavioral test battery. The open field paradigm was found to have the weakest power.

Our meta-analysis further shows that there is no major effect of sex on the behavioral phenotypes, which is in line with the general notion that such differences are also not present in AS patients. We did however find that female wild-type and mutant mice outperformed male wild-type and mutant mice on the rotarod. Improved performance of female mice on the rotarod has also been reported previously [50] and emphasizes the need of using well-matched groups when groups of both sexes of *Ube3a* mice are tested on the rotarod. Given that male mice are heavier than female mice, we investigated if the impaired performance of *Ube3a* mice on the rotarod can be attributed to the increased weight of these mutants. However, we found no correlation between weight of the animal and performance on the rotarod. This observation is in line with other studies [50–52] and indicates that the reduced performance of *Ube3a* mice on the rotarod represents a bona fide impairment in motor performance.

Besides the reproducibility of the observed phenotypes and the high face and construct validity, there are two additional features that make the behavioral test battery for *Ube3a* mice highly useful for drug testing. We show that with the exception of the epilepsy test, all behavioral experiments can be performed with a single cohort of mice, which greatly reduces costs as well as the number of mice needed. In addition, we found that with the exception of the marble burying task, the behavioral test battery can be performed twice with the same cohort while maintaining a phenotype. This makes it possible to test the efficacy of a drug using a within-subject design.

We confirmed previous studies that the audiogenic seizure phenotype is a very powerful test to investigate seizure susceptibility in *Ube3a* mice [7, 13, 18]. With this study, this phenotype is now also confirmed in three independently derived lines: the commonly used *Ube3a*^*tm1Alb*^ line [7], the *Ube3a*^*mSTOP/p+*^ (*Ube3a*^*tm1Yelg*^) line [13], and the recently generated *Ube3a*^mE113X/p+^ (*Ube3a*^*tm2Yelg*^) line [23]. Since nearly all *Ube3a* mice show this phenotype compared to less than 10% of wild-type animals, this test has very high power. Moreover, we showed that the phenotype is readily reversible with the anti-epileptic drug levetiracetam and that the test is highly suitable for dose finding. The only disadvantage of the audiogenic seizure test is that it cannot be performed on the same animals as used in the behavioral test battery, since the sensitivity to audiogenic seizures is exclusively observed in *Ube3a* mice in the 129S2 genetic background.

We also observed an effect of genetic background on the tests of the behavioral test battery. *Ube3a* mice in the C57BL/6J background showed a significant phenotype in the rotarod, nest building, and marble burying tests, but no effect of genotype was observed in the open field test. A significant effect of genotype was found in the forced swim test, but remarkably, this was in the opposite direction. In contrast, *Ube3a* mice in the 129S2 genetic background showed only a significant deficit in the forced swim test (in the same direction as F1 hybrid mice) and no phenotype on any of the other tests of the behavioral battery. This confirms previous reports that many of the *Ube3a* phenotypes are very sensitive to genetic background and not present in 129 lines [8, 9]. There are however several common findings as well as a few discrepancies between these studies and our study. With respect to the rotarod [8, 9] and marble burying phenotype [9], our findings that only *Ube3a-*C57BL/6J and *Ube3a-*F1 hybrid mice show a phenotype are in full agreement with each other (Huang et al. only tested *Ube3a-*C57BL/6J in the marble burying test). With respect to the open field test (distance traveled), the other two studies also found no phenotype in *Ube3a*-129 mice, but in contrast to our findings, they both found a phenotype in *Ube3a-*C57BL/6J mice. One major difference between their and our experimental design is the time the mice were placed in the open field. Indeed, when we left the *Ube3a-*C57BL/6J mice for 30 min in the open field (instead of the 10 min we used), we found a nearly significant phenotype in *Ube3a-*C57BL/6J mice (*p* = 0.06; data not shown). With respect to percentage of time spent in the inner zone of the open field (which is another measure of anxiety), the other two studies showed no significant effect of genotype in any of the genetic backgrounds. Our meta-analysis did however reveal a significant difference between genotypes in F1 hybrid mice (WT 1.1% versus mutant 0.7% time in inner zone; *p* < 0.01), which further indicates that *Ube3a*-mutant mice are more anxious. However, we note that the observed difference was small and a significant effect was only observed in four out of the eight individual experiments. Hence, this measure is not very robust.

Taken all studies into consideration, it is clear that *Ube3a* mice in the F1 hybrid 129S2-C57BL/6J background show the most robust phenotypes, with the notable exception of the audiogenic seizure susceptibility test, which is strictly seen in *Ube3a*-129S2 mice. The question arises whether the observed differences between *Ube3a* mice in different genetic backgrounds have any translational significance. The lack of phenotypes of *Ube3a*-129S2 mice in most tests could simply reflect the passive/hypoactive phenotype of these mice, resulting in a floor effect. However, it could also be that the AS phenotype is sensitive to genetic background and that the changes that are observed between individuals with AS are in part caused by genetic modifiers, rather than the nature of the mutation. Detailed studies of individuals with recurrent or similar mutations could provide more insight in that question [53].

To test the translational value of the behavioral test battery, we decided to re-evaluate the two drugs that previously were tested in clinical trials involving individuals with AS: minocycline (trial register NCT01531582 [20] and NCT02056665 [22]) and levodopa (trial register NCT01281475 [21]). Both drugs were previously shown to rescue the rotarod impairment of *Ube3a* mice (see NCT01531582 for minocycline, and [21] for levodopa). In addition, minocycline rescued the hippocampal LTP deficit of *Ube3a* mice [20], whereas levodopa rescued the increased phosphorylation of CaMK2 observed in *Ube3a* mice [21]. We tested the effect of both drugs on all tests of our behavioral test battery, using the same drug administration protocols as used for the original studies. In addition, we also tested the effect of minocycline when administered from birth, as previously published for the Fragile X mouse model [26]. However, in line with the clinical trials, we did not observe any efficacy of these drugs when tested on *Ube3a* mice. Our finding that minocycline and levodopa are unable to improve performance on the rotarod is at odds with aforementioned previous preclinical studies. Failure of replication could be due to differences in strains or procedures, although there is full agreement between our labs with respect to performance of *Ube3a* mice on the rotarod and the effects of different genetic backgrounds on this performance [9]. We think it is more likely that the rotarod experiments used for the preclinical studies were underpowered, as our analysis showed that 14 mice per group are needed for a well-powered rotarod study using two groups. In the levodopa study, the authors used 6 different treatment groups and only 6 mice per group [21]. Such small sample sizes make the test underpowered and also very vulnerable for the sex differences that we describe here. Since the details of the rotarod experiments of the minocycline treatment were not provided (NCT01531582), we cannot comment on these discrepancies.

## Conclusions

Here, we provided a behavioral test battery with a robust set of well-characterized *Ube3a* phenotypes, which allows researchers to investigate the effects of pharmacological and genetic interventions involving *Ube3a* mice. A standardized set of tests, in combination with a well-defined genetic background, will also be very useful to compare data across laboratories. Moreover, using a standardized behavioral test battery may reduce selective reporting bias [54]. Future studies should reveal how well the results of this behavioral test battery can be replicated between different laboratories in which housing and testing environment is different [55–58]. In addition, robust tests that capture phenotypes in the domain of cognitive function should be identified and added to this test battery.

## Abbreviations

AS: Angelman syndrome
EEG: Electroencephalography
IP: Intraperitoneal
Mut: Mutant
UBE3A: Ubiquitin-protein ligase E3A
WT: Wild-type
